# Compartment-specific dynamics of soil microbiota along a *Pinus armandii* plantation chronosequence in karst mountain ecosystems

**DOI:** 10.3389/fmicb.2025.1626892

**Published:** 2025-07-01

**Authors:** Bin He, Ping Zhang, Xiaolong Bai, Wangjun Li, Shun Zou

**Affiliations:** ^1^College of Ecological Engineering, Guizhou University of Engineering Science, Bijie, China; ^2^Guizhou Province Key Laboratory of Ecological Protection and Restoration of Typical Plateau Wetlands, Bijie, China

**Keywords:** karst mountain ecosystems, soil microbial community, compartment-specific dynamics, stand age chronosequence, plantation

## Abstract

Soil microbiomes play pivotal roles in mediating plant diversity maintenance by regulating multifunctional ecosystem services during plant development. However, how different stand age of plants influence soil microbial communities in various soil compartments remains poorly understood. Through Illumina-based 16S rRNA and ITS amplicon sequencing, we systematically investigated the successional trajectories of soil microbiome in *Pinus armandii* plantations spanning various developmental phases. Key findings revealed that stand age exerted a stronger influence on microbial restructuring than soil compartment, significantly altering community composition in both soil types. Alpha diversity (Shannon and Chao1 indices) exhibited a U-shaped trajectory with stand age, except for fungal Chao1 in bulk soil. While dominant bacterial and fungal phyla remained relatively stable, community composition displayed significant stage-dependent variations. Co-occurrence network analysis demonstrated lower fungal network complexity compared to bacterial networks, with rhizosphere soils harboring more intricate interactions compared to bulk soils. Community assembly mechanisms diverged: deterministic processes dominated bacterial assembly, whereas stochasticity governed fungal communities. Soil properties exerted significant influences on microbial composition and diversity: bacterial composition correlated strongly with pH and stoichiometric ratios (C/N, C/P, N/P), while fungal composition showed stronger associations with TN, TP, and AN. Our results demonstrate that *P. armandii* plantations maintain core phylum-level microbial populations while developing stage-specific diversity patterns. Crucially, bacteria and fungi exhibit divergent responses to stand development, highlighting their divergent ecological strategies in adapting to nutrient-limited karst ecosystems.

## 1 Introduction

The karst-dominated southwestern region of China, characterized by complex geomorphology and significant climatic heterogeneity, is a globally recognized biodiversity hotspot (Myers et al., 2000), harboring rich rare species and serving as both a vital genetic reservoir and a key ecological barrier for the Yangtze River basin. However, extensive logging since the mid-20th century, followed by large-scale artificial afforestation, has degraded the landscape. While alleviating some ecological pressures, these plantations now face severe challenges—including declining soil nutrients, reduced water conservation capacity, and difficulties in natural seedling regeneration—primarily due to early practices of high-density monoculture planting. This degradation compromises ecosystem stability and service provision, making the enhancement of functional quality and ecological sustainability in these degraded plantations within the fragile karst environment a critical scientific and practical priority.

As fundamental constituents of soil ecosystems, soil microbial communities demonstrate remarkable biodiversity and functional complexity, constituting critical mediators in soil–plant interactions. These microorganisms play pivotal roles in modulating biogeochemical cycles and sustaining ecosystem functionality throughout ecological restoration processes (Delgado-Baquerizo et al., 2020). Soil microbes are not only key drivers of nutrient cycling, extensively participating in many ecological processes such as biological nitrogen fixation, organic phosphorus mineralization, and organic carbon assimilation (Muhammad et al., 2021), but also serve as dynamic nutrient reservoirs modulating soil fertility dynamics. The composition of soil microbiota exhibits pronounced sensitivity to environmental fluctuations, with distinct community assemblages developing across spatial gradients and temporal sequences (Jiang et al., 2018). Thus, understanding the microbial community dynamics, the factors driving these changes, and the underlying mechanisms is a central issue in ecological restoration research (Liu L. et al., 2020). Insights into microbial community dynamics during ecological restoration are critical for maintaining ecosystem services, supporting sustainable development, formulating effective management strategies, and preventing soil-borne diseases (Li et al., 2023).

Microbial communities exhibit distinct biogeographical patterns across spatial scales, with resource availability acting as a primary constraint (Ji et al., 2020). These spatial distributions arise from complex interplay between species interactions, dispersal constraints, and environmental filtering (Jiao et al., 2021). Particularly noteworthy is the rhizosphere microenvironment, where plant root exudates mediate dynamic plant-microbe interactions through continuous release of carbon substrates, secondary metabolites, and signaling molecules that drive co-evolutionary adaptations and mutualistic associations (Bakker et al., 2015; Walters et al., 2018). This chemically enriched zone sustains 2–3 times higher microbial biomass than bulk soil (Hartmann et al., 2009), stimulating enhanced enzymatic activity and nutrient transformation rates (Bakker et al., 2013). Remarkably, this rhizosphere effect creates divergent microbial functional profiles and nutrient transformation rates between root-associated and bulk soil compartments (Bakker et al., 2015). Despite these functional distinctions, current soil microbial ecology paradigms predominantly derive from bulk soil analyses (Shen et al., 2013; Li D. D. et al., 2018; Li J. et al., 2018), while rhizosphere dynamics remain comparatively understudied. Particular knowledge gaps exist regarding temporal variations in rhizosphere-bulk soil differentiation patterns across stand ages (LeBauer and Treseder, 2008; Yue et al., 2017).

The composition and diversity of soil microbial communities are shaped by an intricate interplay of biotic and abiotic drivers. Biotic regulators include vegetation characteristics (Feng and Wang, 2023), while abiotic controls encompass temperature gradients (Cavicchioli et al., 2019), soil pH (Rousk et al., 2010), nitrogen availability (Cederlund et al., 2014), and organic carbon dynamics (Sul et al., 2013). Differential environmental responses among microbial taxa (bacteria, archaea, fungi) drive spatial distribution patterns and community assembly mechanisms (Zhang et al., 2017). Although soil physicochemical properties are well-established determinants of microbial assemblage (Wang et al., 2017; Ni et al., 2021), the functional linkage between vegetation traits and forest soil microbiota remains contentious (Huo et al., 2023). Plant communities mediate microbial composition through three primary pathways: (1) direct host–microbe interactions in the rhizosphere (Martínez-García et al., 2015), (2) indirect modulation of soil properties (Zak et al., 2003), and (3) coupled plant–soil feedbacks that jointly regulate microbial dynamics (Bai et al., 2019; Landesman et al., 2014). Such interactions drive spatiotemporal reorganization of soil biota through plant-mediated alterations in ecological processes (Ward et al., 2015; Yao et al., 2018), with plant species identity (Gao et al., 2015) and community diversity (Liu W. et al., 2020) emerging as critical determinants across ecosystems. Additionally, plant stand age are critical in shaping underground microbial communities, as different stages of plant growth release can distinctly root exudates, which influence microbial dynamics and, in turn, plant growth (Zhao et al., 2021). As a result, these interactions lead to differentiated soil microbial communities across various ecological niches. Preliminary studies suggest that microbial communities, including bacterial and fungal species (Wattenburger et al., 2019), exhibit variations across different plant growth stages. Despite advances in understanding forest soil microbiomes (Paula et al., 2014), critical knowledge gaps persist regarding the synergistic effects of ecological niches, edaphic factors, and stand age on microbial community assembly (Zhou and Ning, 2017).

*Pinus armandii*, an endemic evergreen conifer predominantly distributed across central and western China, serves as a keystone species in ecological restoration initiatives within the fragile karst landscapes of southwest China. Its ecological significance extends to safeguarding soil stability and sustaining agroforestry systems in this ecologically vulnerable region (Yao et al., 2021). Currently, research on *Pinus armandii* has expanded to cover various aspects, including community structure, succession trends, pest control, seedling cultivation, genetic improvement, and plant diversity (Li et al., 2024). However, studies on its adaptability in fragile karst ecosystems, particularly concerning soil microbial community responses to stand age gradients, remain limited. This gap impedes the broader application of *Pinus armandii* in combating desertification and restoring degraded karst ecosystems. To address this deficiency, we conducted a comparative microbiome analysis of rhizosphere and bulk soil microbiomes in *P. armandii* plantations of varying stand age within karst topography using Illumina-based 16S rRNA and ITS sequencing. We hypothesized that (1) niche-based selection drives significant divergence in microbial composition and metabolic potential between rhizosphere and bulk soils, with rhizosphere bacteria and fungi being more sensitive to soil properties; and (2) microbial community assembly follows phasic successional patterns across stand age, with mature forests exhibiting greater functional stability and mutualistic interactions. Our study aims to elucidate: (i) niche differentiation in microbial structure and putative metabolic functions; (ii) temporal dynamics of bacterial/fungal assemblages across stand age; and (iii) key environmental drivers governing these successions. These findings will clarify plant-microbe interactions during long-term karst vegetation restoration, providing critical insights for sustainable subalpine plantation management and soil fertility preservation in Southwest China.

## 2 Materials and methods

### 2.1 Study site and experimental design

The study was conducted in the dominant distribution range of *Pinus armandii* plantations in Bijie City (26°21’N-27°46’N, 103°36’E-106°43’E), Guizhou Province, China. This region is characterized by a subtropical humid monsoon climate, with altitudes ranging from 457 to 2910.3 m, average annual precipitation between 849 and 1,399 mm, and average temperatures ranging from 10°C to 15°C.

We investigated mono-specific *P. armandii* plantations at three successional stages (young, middle-aged, mature) with homogeneous geomorphic conditions in August 2021 during peak vegetation growth. A nested sampling design was implemented with three replicate 20 × 20 m plots per stand age. Plots were intentionally spaced ≥150 m apart to minimize spatial autocorrelation and ensure independence (Mariotte et al., 1997). Plot selection adhered to national forest resource survey protocols for age classification (GB/T 26424–2010). Within each plot, all trees with DBH > 3 cm (diameter at breast height, 1.3 m) were measured for dendrometric parameters (height, DBH, crown width). Understory vegetation was quantified through five randomly positioned shrub (2 × 2 m) and herbaceous (1 × 1 m) subplots per main plot, recording species composition and structural parameters (density, coverage, vertical stratification).

Rhizosphere and bulk soils were systematically collected using compartment-specific techniques with explicit replication at multiple spatial scales to ensure representativeness and analytical robustness.

#### 2.1.1 Rhizosphere soil

Within each main plot (*n* = 3 per age), five healthy trees were randomly selected. From each selected tree, rhizosphere soil (soil adhering to fine roots after gentle brushing) was collected from three equidistant points around the root zone. Soil from all five trees per plot (15 collection points) was composited into one representative rhizosphere soil sample per plot, yielding 3 composite rhizosphere samples per stand age (Philippot et al., 2013).

#### 2.1.2 Bulk soil

Within each main plot (*n* = 3 per age), bulk soil was collected using five individual cores (20-cm depth) arranged in a standardized S-pattern to cover the plot area representatively. A > 2.5 m buffer from plot edges was maintained. Soil from these five cores per plot was composited into one representative bulk soil sample per plot, yielding 3 composite bulk soil samples per stand age.

This nested design resulted in a total of 18 composite soil samples (3 stand ages × 3 replicate plots × 2 soil compartments). All samples were immediately stored in sterile bags, transported on ice, and processed for bifurcated preservation. One subsample was cryopreserved (−80°C) for molecular analyses, while the air-dried counterpart served (2 mm mesh) for physicochemical characterization. Samples were coded using a two-letter system: initial letter for soil type (B: bulk; R: rhizosphere), secondary letter for stand age (Y: young; M: middle-aged; O: mature).

### 2.2 Soil physicochemical characterization

Soil pH was determined potentiometrically in 1:2.5 (w/v) soil-water suspensions using a calibrated pH meter (InsMark^™^ IS126, Shanghai, China). Total carbon (TC) quantification employed high-temperature combustion with an Elementar TOC analyzer (Hanau, Germany). Soil organic carbon (SOC) was quantified via dichromate oxidation-external heating (Walkley-Black modified method). Nitrogen fractions were analyzed through: (1) total nitrogen (TN) by micro-Kjeldahl digestion, (2) available nitrogen (AN) via alkaline diffusion. Potassium speciation included: (1) total potassium (TK) by atomic absorption flame photometry, (2) available potassium (AK) through ammonium acetate extraction. Phosphorus fractions were determined calorimetrically using molybdenum antimony anti-colorimetric method for both total (TP) and available phosphorus (AP). All analyses followed standardized procedures (Bao, 2000).

### 2.3 Microbial community profiling

Total DNA was extracted from both bulk soil and rhizosphere samples using the E.Z.N.A^™^ Mag-Bind Soil DNA Kit (OMEGA) according to the manufacturer’s instructions. Hypervariable regions were amplified with proofreading polymerase: bacterial 16S rRNA V3-V4 using 515F/806R primers and fungal ITS1-5F with ITS5-1737F/ITS2-2043R. PCR reactions (25 μL) contained: 2 μL template DNA, 5X buffer, 2.5 mM dNTPs, 0.25 μL HiFi polymerase, and 1 μL primers. Thermal cycling conditions:

Bacteria: 98°C/1 min; 30 cycles of 98°C/10s, 50°C/30s, 72°C/30s;
Fungi: 98°C/2 min; 30 cycles of 98°C/15 s, 55°C/30s, 72°C/30s; final extension 72°C/30s.

Purified amplicons (Qiagen Gel Extraction Kit) were sequenced on Illumina NovaSeq (2 × 250 bp) at Novogene (Beijing).

### 2.4 Bioinformatic processing

Raw sequences were demultiplexed in QIIME2 (2020.06) and processed through DADA2 (v1.8) for quality filtering, paired-end merging, and ASV clustering. Taxonomic assignment used SILVA v132 (bacteria) and UNITE v8.0 (fungi) databases with 97% similarity thresholds. Rarefaction to 1,067,590 bacterial and 1,095,406 fungal sequences ensured uniform analysis. Data are deposited in NCBI SRA (PRJNA1063200, PRJNA1132892).

### 2.5 Statistical analyses

To elucidate microbial interaction patterns across the chronosequence and soil compartments, bacterial and fungal co-occurrence networks were generated through Sparse Correlations for Compositional data (SparCC). Spearman’s rank correlation analysis of bacterial and fungal ASVs abundance tables yielded correlation coefficients (R) and significance values (P) (Barberán et al., 2012). Network topology was defined using adjacency matrices derived from correlation coefficients, with edge significance thresholds established through random matrix theory (RMT). Final network visualizations were rendered using Gephi v0.9.2.

To quantify assembly processes, we applied null model-based phylogenetic *β*-diversity analysis using the picante R package. The β-Nearest Taxon Index (β-NTI) values were derived from 999 randomizations of the observed ASV table and phylogenetic tree. |β-NTI| > 2 indicated deterministic processes (heterogeneous selection for β-NTI > 2; homogeneous selection for β-NTI < −2), while |β-NTI| < 2 indicated stochastic processes (Stegen et al., 2012). For stochastic processes, the contribution of dispersal limitation (RCBray > 0.95), homogenizing dispersal (RCBray < −0.95), and ecological drift (|RCBray| < 0.95) was further quantified using the RCBray value (Stegen et al., 2013).

Alpha diversity indices (Chao1 and Shannon) were calculated using the sequencing company’s analysis platform. Functional classifications of microbial communities were predicted using PICRUSt for bacteria and FUNGuild for fungi (Langille et al., 2013). Parametric comparisons were conducted through one-way ANOVA with post-hoc LSD tests for multi-group comparisons, supplemented by independent t-tests for pairwise chronosequence and soil compartment analyses. Microbial community structure was ordinated via principal coordinate analysis (PCoA) with Bray–Curtis dissimilarity matrices. Mantel tests (MT) quantified correlations between soil physicochemical parameters and alpha diversity metrics. Taxon-environment relationships were elucidated through redundancy analysis (RDA) implemented in Canoco 5.0. All statistical procedures were executed in SPSS 23 (IBM Corp.) and R v3.5.3, with significance thresholds defined at *p < 0.05*.

## 3 Results

### 3.1 Microbial diversity

**Figure 1:**
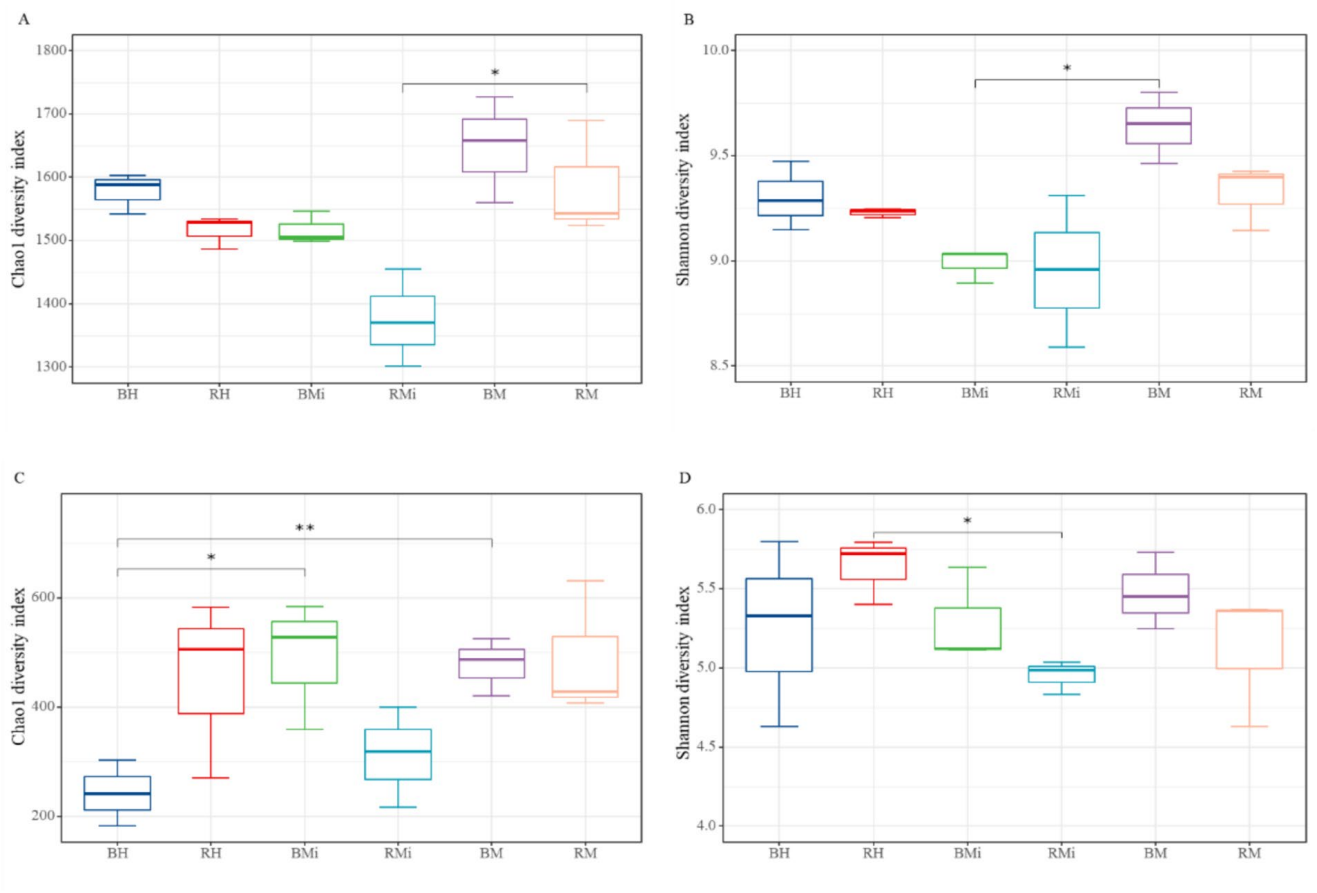
Differences in bacterial **(A,B)** and fungal **(C,D)** alpha diversity between rhizosphere and bulk soil along the age gradient: **(A)** changes of bacterial Chao 1 diversity in rhizosphere and bulk soil; **(B)** changes of bacterial Shannon diversity in rhizosphere and bulk soil; **(C)** changes of fungal Chao 1 diversity in rhizosphere and bulk soil; **(D)** changes of fungal Shannon diversity in rhizosphere and bulk soil. Any statistically significant differences among the soil samples are denoted with an asterisk (** and * represent *p <* 0.01, and 0.05 according to T-test, respectively).

Alpha diversity (Shannon and Chao1 indices) varied significantly across stand age and soil niches (Figure 1). In middle-aged and mature forests, rhizosphere soil exhibited lower bacterial and fungal alpha diversity than bulk soil. However, in young forests, rhizosphere soil showed lower bacterial but higher fungal alpha diversity. Overall, significant differences in alpha diversity were observed across the chronosequence for both soil compartments (*p < 0.05*), generally showing an initial decline followed by an increase with increasing stand age, except for fungal Chao1 diversity in bulk soil.

**Figure 2:**
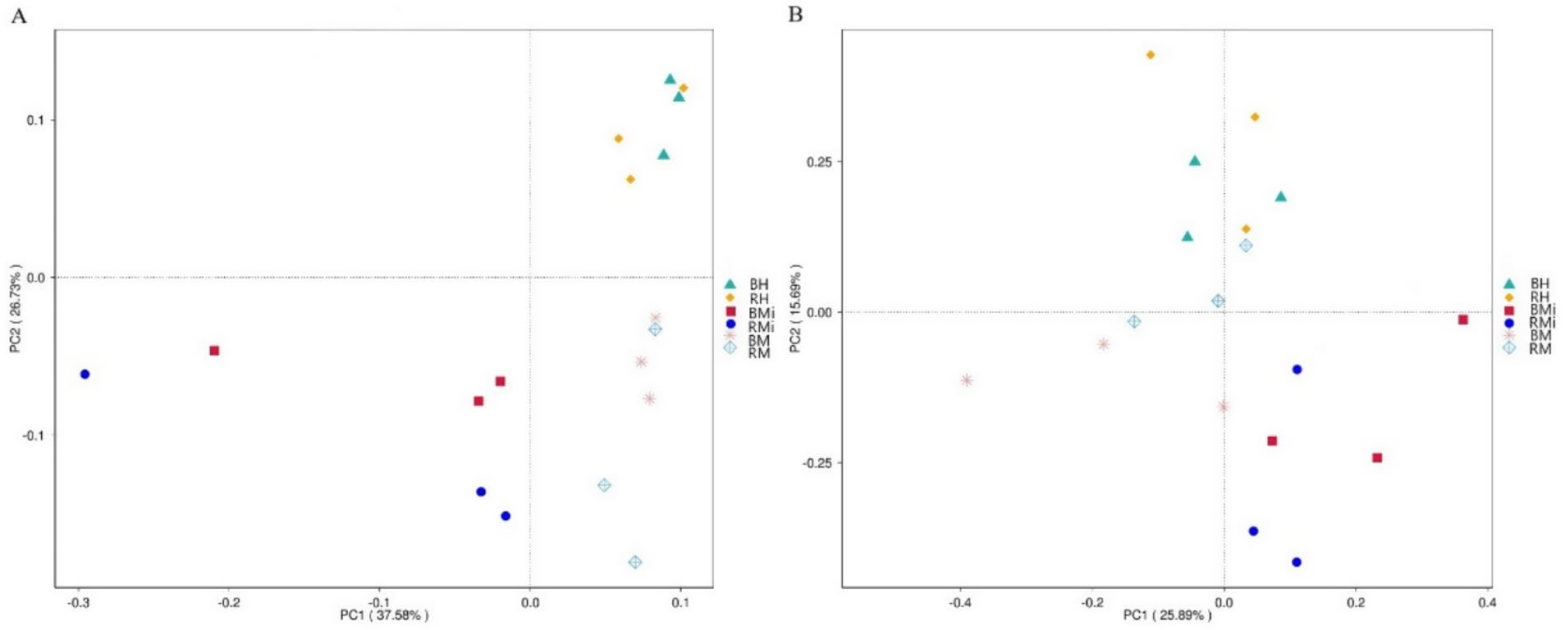
Principal coordinate analysis (PCoA) of soil bacterial **(A)** and fungal **(B)** community composition based on Bray-Curtis distance between bulk and rhizosphere soils under different stand age.

Beta diversity analysis using Principal Coordinates Analysis (PCoA) revealed distinct bacterial community structures across stand age in both soil compartments (Figure 2A). Bulk soil showed more pronounced changes (*p* < 0.001; *R^2^* = 0.66) than rhizosphere soil (*p* < 0.001; *R^2^* = 0.59). Fungal community structure varied spatially; bulk soil communities separated along the second principal coordinate, while rhizosphere communities separated along the first (Figure 2B). PCoA based on Bray–Curtis similarity confirmed significant differences in fungal community composition across stand age in both bulk (*p* < 0.001, *R^2^* = 0.67) and rhizosphere (*p* < 0.001, *R^2^* = 0.64) soils.

### 3.2 Taxonomic composition

**Figure 3:**
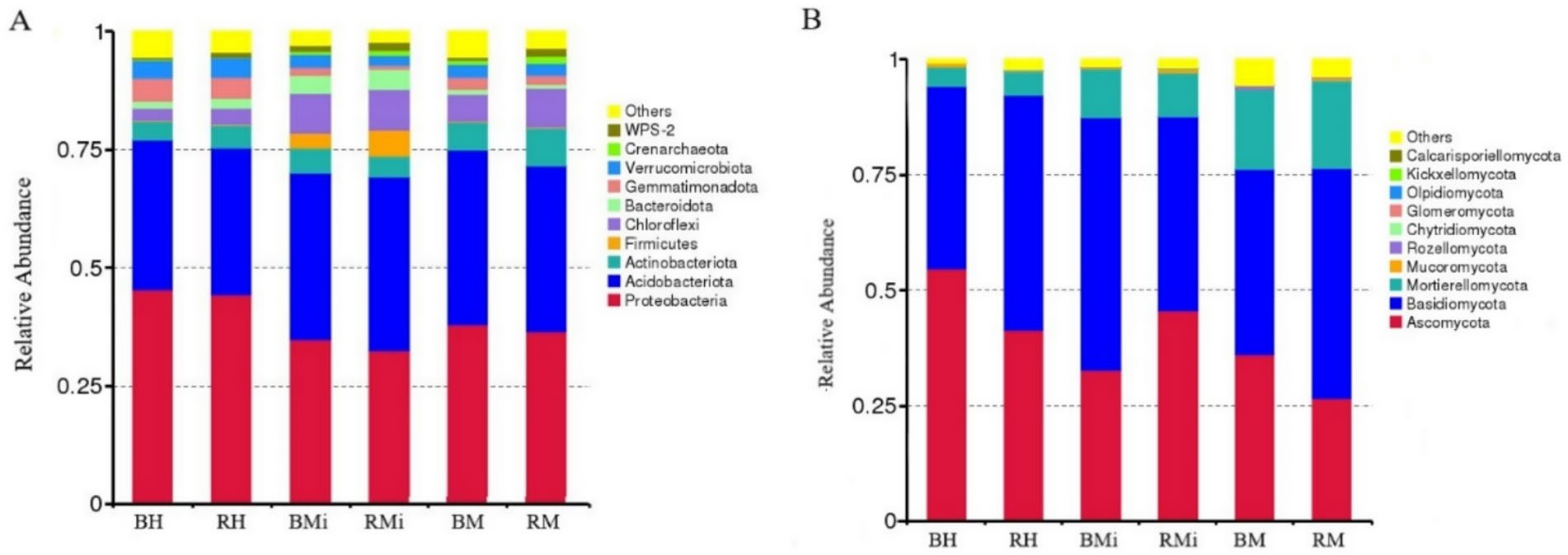
Relative abundance of the dominant bacterial and fungal taxa among different stand age. **(A)** Relative abundance of bacterial phyla (%), **(B)** relative abundance of fungal phyla (%).

**Figure 4:**
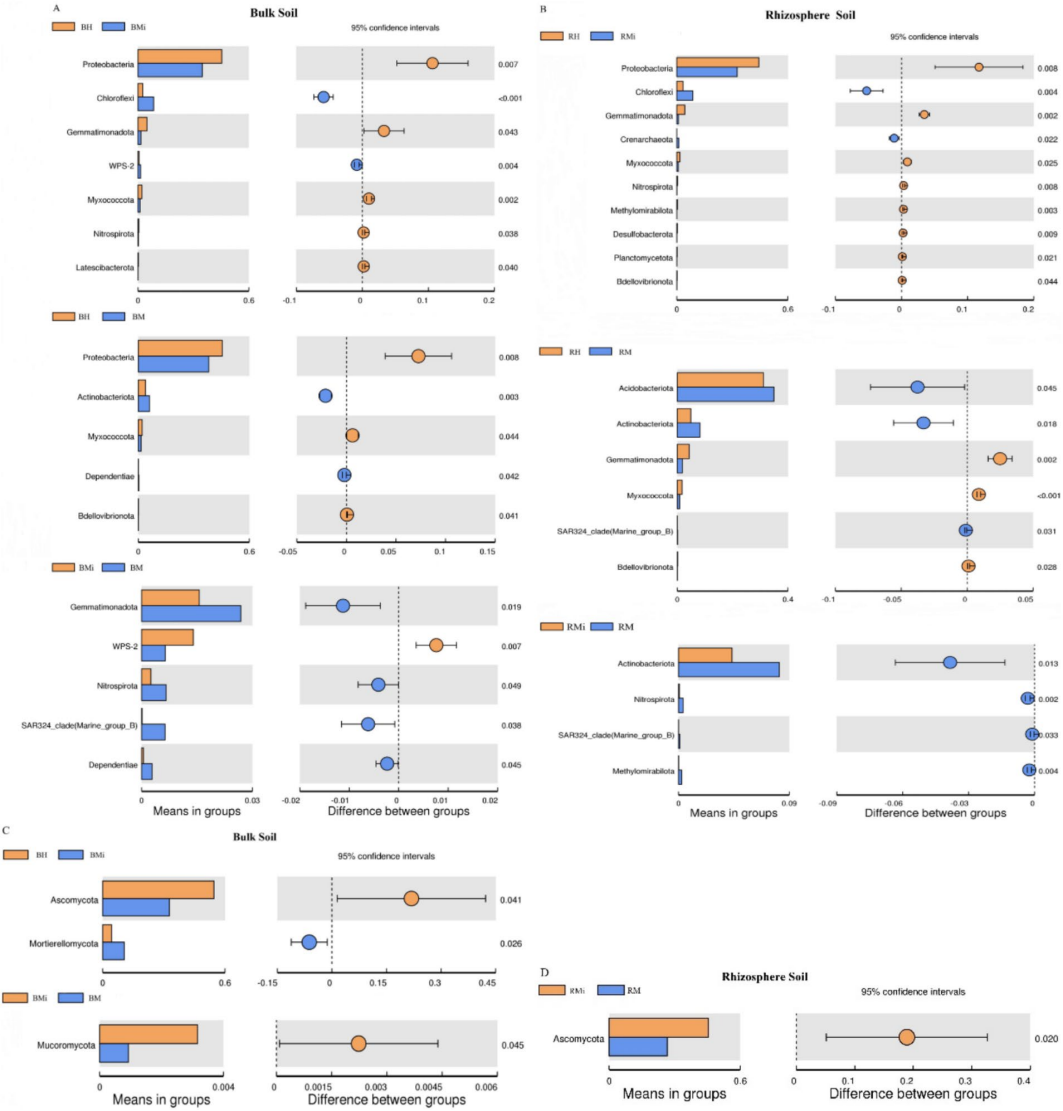
T-test analysis of bacterial and fungal communities in both the bulk soil and rhizosphere soil. **(A,B)** Represent the difference of bacterial composition among different stand ages in bulk soil and rhizosphere soil, respectively; **(C,D)** represent the difference of fungal composition among different stand ages in bulk soil and rhizosphere soil, respectively.

Taxonomic analysis at the phylum level revealed consistent dominant bacterial phyla (Proteobacteria, Acidobacteria, Actinobacteria, and Chloroflexi) across soil compartments, accounting for approximately 85% of the total relative abundance (Figure 3A). However, their relative abundances varied significantly across stand age and soil niches (Figures 4A,B; *p < 0.05*). In bulk soil, Proteobacteria decreased while Chloroflexi increased in middle-aged forests. Conversely, Acidobacteria and Actinobacteria showed a gradual increase across the chronosequence. In rhizosphere soils, Acidobacteria and Chloroflexi peaked in the middle-aged forest, while Proteobacteria and Actinobacteria displayed a declining trend. While Proteobacteria were more abundant in bulk soil and Chloroflexi in rhizosphere soil, these differences were not statistically significant (*p > 0.05*).

Similarly, the dominant fungal phyla (Ascomycota, Basidiomycota, and Mortierellomycota) comprised approximately 97% of the total relative abundance (Figure 3B). Their relative abundances varied across stand age and soil niches (Figures 4C,D), although significant differences between rhizosphere and bulk soil were not observed (*p > 0.05*). Basidiomycota abundance was lowest in middle-aged rhizosphere soil, while Ascomycota peaked at this stage. The opposite trend was observed in bulk soil. Mortierellomycota abundance increased consistently across the chronosequence.

### 3.3 Microbial community networks

**Figure 5:**
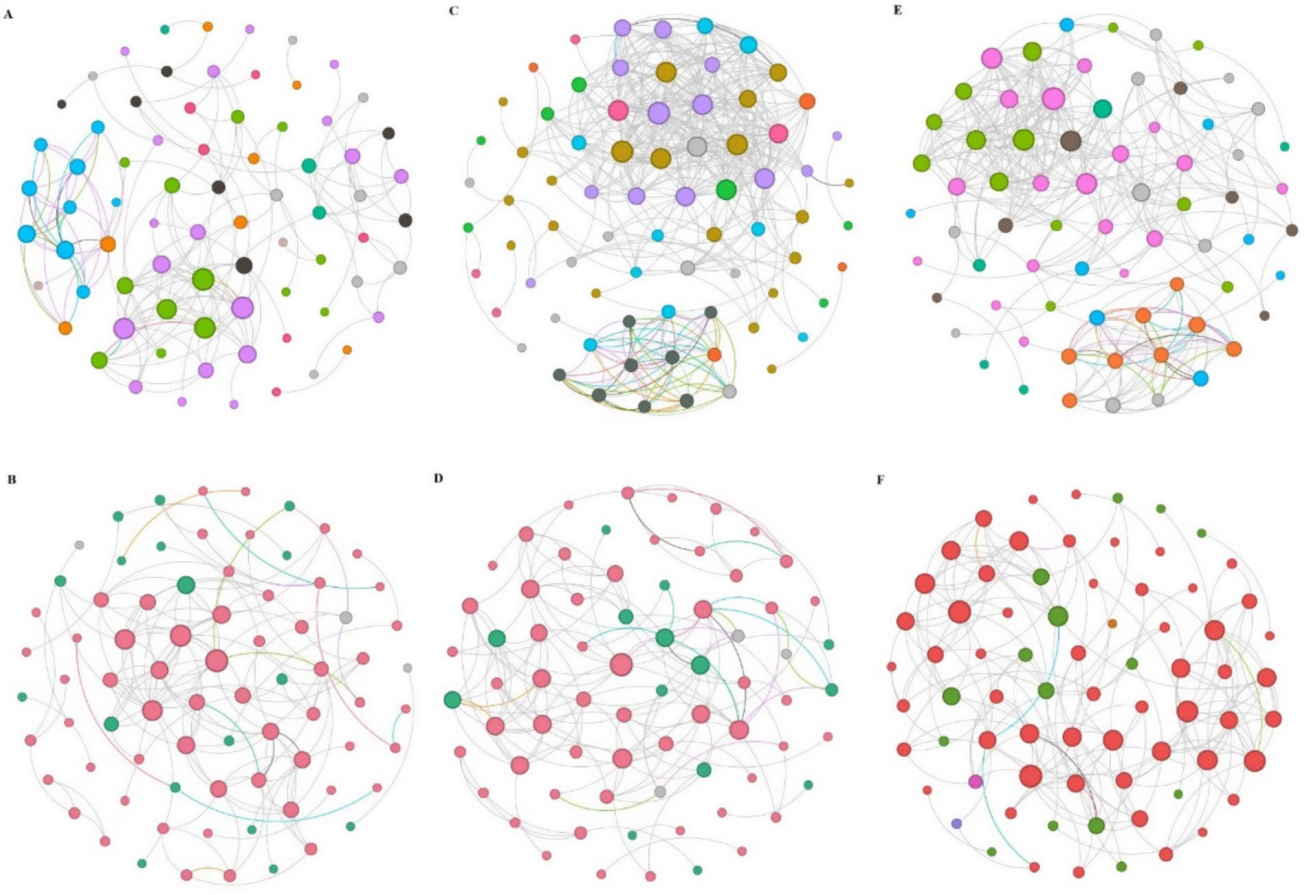
Bacterial and fungal co-occurrence networks across the chronosequence and soil compartment. **(A,B)** Represent the co-occurrence network of bacteria and fungi in the bulk soil, respectively. **(C,D)** Represent the co-occurrence network of bacteria and fungi in the rhizosphere soil, respectively. **(E,F)** Represent bacterial and fungal co-occurrence patterns between the bulk and rhizosphere soil.

Network analysis revealed significant differences in microbial co-occurrence patterns between rhizosphere and bulk soils across all stand ages (Figures 5A,C; Supplementary Table S1). Bacterial networks in rhizosphere soil were larger and more connected than those in bulk soil, with more nodes, a higher proportion of positive edges and greater average connectivity. However, rhizosphere networks had lower diameter, modularity, and average path length. No network hubs were detected in either soil type, although 10 and 12 module hubs were observed in rhizosphere and bulk soil networks, respectively. Keystone taxa were identified in both soil types, with Proteobacteria and Actinobacteria consistently prominent. The top seven keystone species in rhizosphere soil networks included Proteobacteria (26.67%), Actinobacteria (17.33%), Bacteroidota (12%), Firmicutes (10.67%), Chloroflexi (9.33%), Acidobacteria (6.67%), and Verrucomicrobiota (5.33%). In contrast, the bulk soil networks were dominated by Proteobacteria (25.68%), Actinobacteria (17.57%), Firmicutes (12.16%), Chloroflexi (9.46%), Bacteroidota (9.46%), Acidobacteria (6.67%), and Myxococcus (4.05%).

Fungal networks in rhizosphere soil showed a larger proportion of positive edges, higher average connectivity, average cluster coefficient, density, and modularity compared to bulk soil networks (Figures 5B,D; Supplementary Table S1). No network hubs were identified in either soil type, but a greater number of module hubs was observed in bulk soil (15) compared to rhizosphere soil (11). The top five keystone species in bulk soil networks included Ascomycota (73.68%), Basidiomycota (22.37%), Chytridiomycota (1.32%), Mucoromycota (1.32%), and Olpidiomycota (1.32%). In rhizosphere networks, the dominant taxa were Ascomycota (75.71%), Basidiomycota (20%), Chytridiomycota (1.43%), Glomeromycota (1.43%), and Olpidiomycota (1.43%). Overall, bacterial networks exhibited greater complexity than fungal networks, as indicated by multiple topological properties (Figures 5E,F; Supplementary Table S2).

### 3.4 Soil microbial community assembly

**Figure 6:**
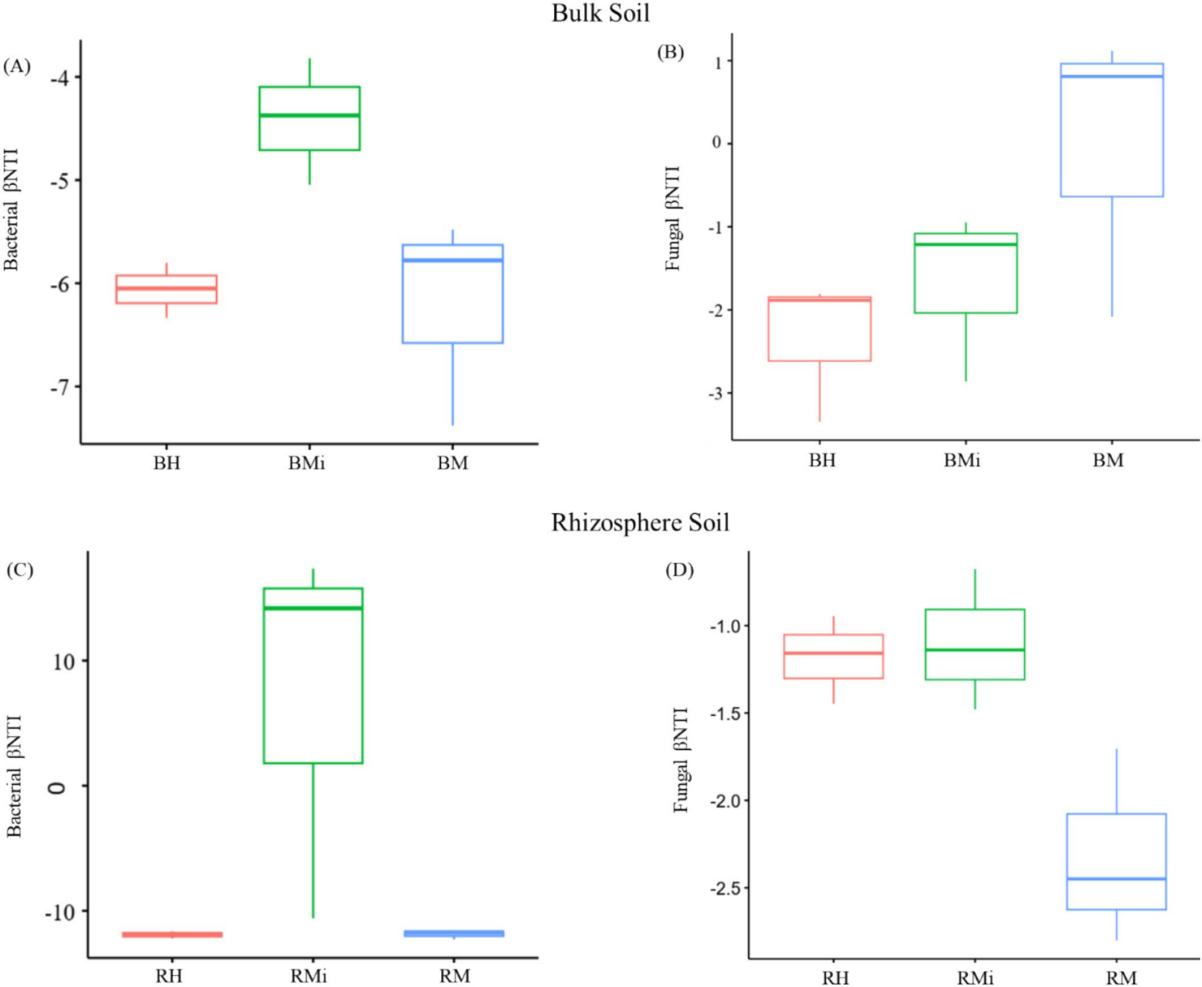
Assembly processes of the soil microbial community across the chronosequence and soil compartment. **(A)** represent the assembly processes of bacterial communities in the bulk soil, **(B)** represent the assembly processes of fungal communities in the bulk soil, **(C)** represent the assembly processes of bacterial communities in the rhizosphere soil, **(D)** represent the assembly processes of fungal communities in the rhizosphere soil.

Null model analysis revealed distinct stand age-dependent variations in the relative contributions of stochastic and deterministic processes to microbial community assembly (Figure 6). Bacterial communities were predominantly structured by deterministic processes across all stand ages. Notably, stochasticity exerted a stronger influence on bacterial composition in rhizosphere soils than in bulk soils (Figures 6A,C). Conversely, fungal community assembly was primarily driven by stochastic mechanisms. A critical transition was observed in rhizosphere fungal communities, where the dominant assembly process shifted from stochastic to deterministic during forest development (Figures 6B,D).

### 3.5 Predicted microbial functions

**Figure 7:**
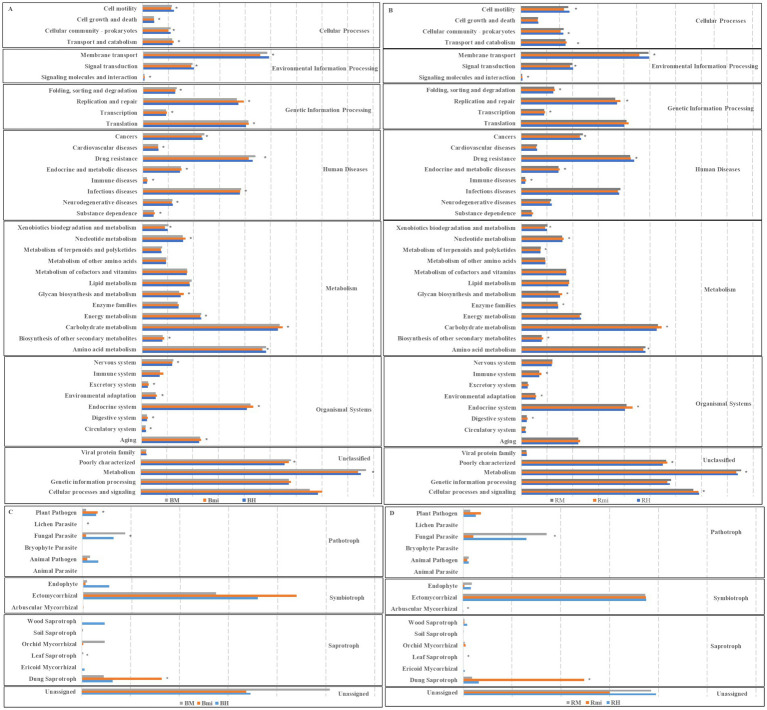
Predicted functions of soil microbial community among different successional stages. **(A)** Functional group of bacterial communities in bulk soils. **(B)** Functional group of bacterial communities in rhizosphere soils. **(C)** Functional group of fungal communities in bulk soils. **(D)** Functional group of fungal communities in rhizosphere soils. Asterisks (∗*p < 0.05*) indicate significant differences between successional stages.

PICRUSt analysis of bacterial communities revealed 44 pathways, predominantly associated with environmental information processing, genetic information processing, and metabolic pathways (Figures 7A,B). While no significant differences in the relative abundance of bacterial functional categories were observed between rhizosphere and bulk soils, 35 and 28 pathways showed substantial changes (*p < 0.05*) across stand age for bacterial communities in bulk and rhizosphere soils, respectively.

FUNGuild analysis of fungal communities revealed three trophic modes (pathotroph, symbiotroph, and saprotroph), with rhizosphere soil exhibiting higher relative abundances of pathotrophic functions (Figures 7C,D). Significant changes in specific fungal functional groups were observed across stand age in both soil compartments (*p < 0.05*). Specifically, in bulk soils, the relative abundance of plant pathogens, fungal parasites, lichen parasites, leaf saprotrophs, and dung saprotrophs significantly changed (*p < 0.05*) with stand age. In rhizosphere soils, significant changes (*p < 0.05*) were observed in fungal parasites, arbuscular mycorrhizal fungi, leaf saprotrophs, and dung saprotrophs.

### 3.6 Environmental drivers of microbial community

**Figure 8:**
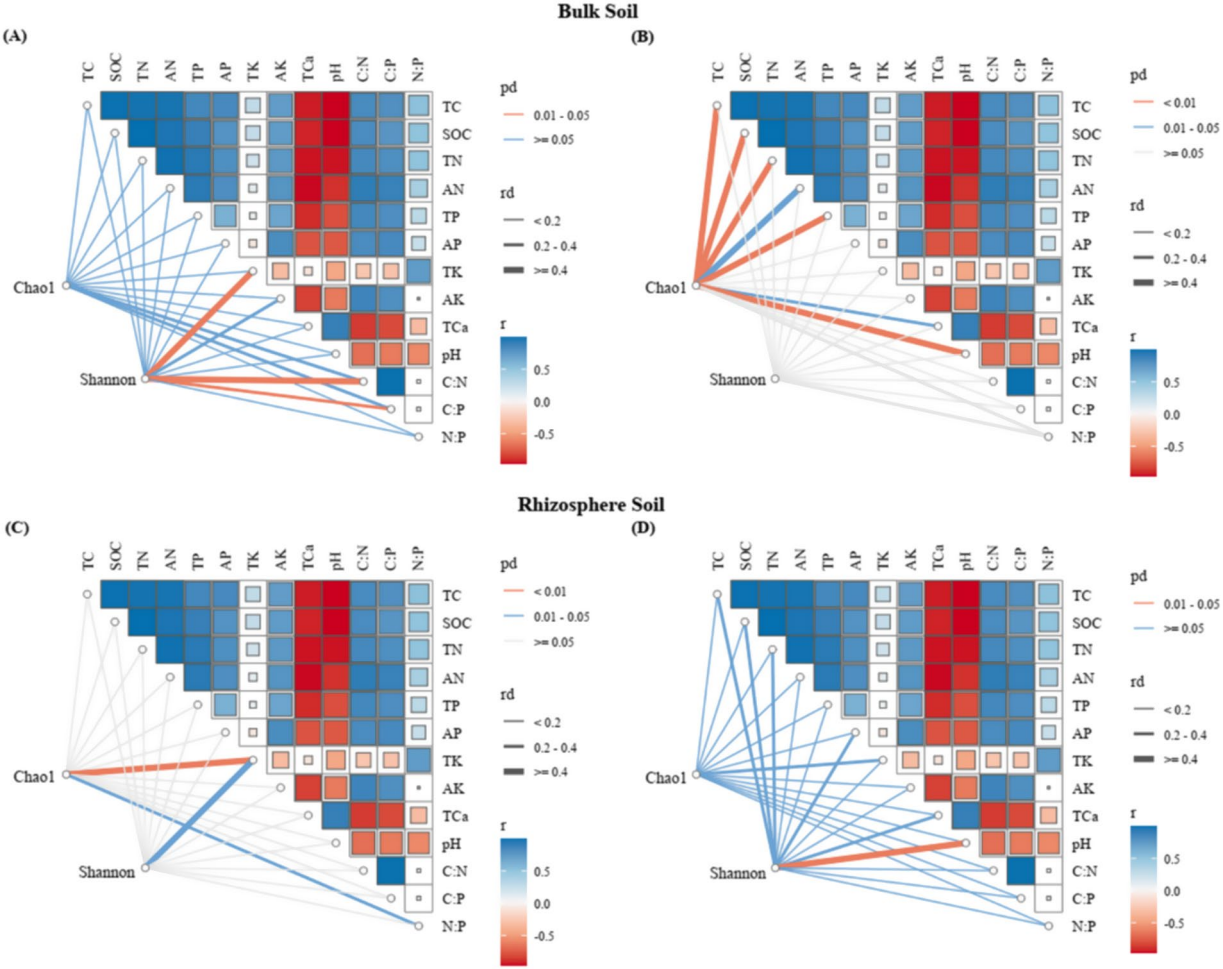
Mantel test (MT) analyzed the relationship between soil characteristics and microbial diversity for different niche compartments (**A,C**: bacteria; **B,D**: fungi).

**Figure 9:**
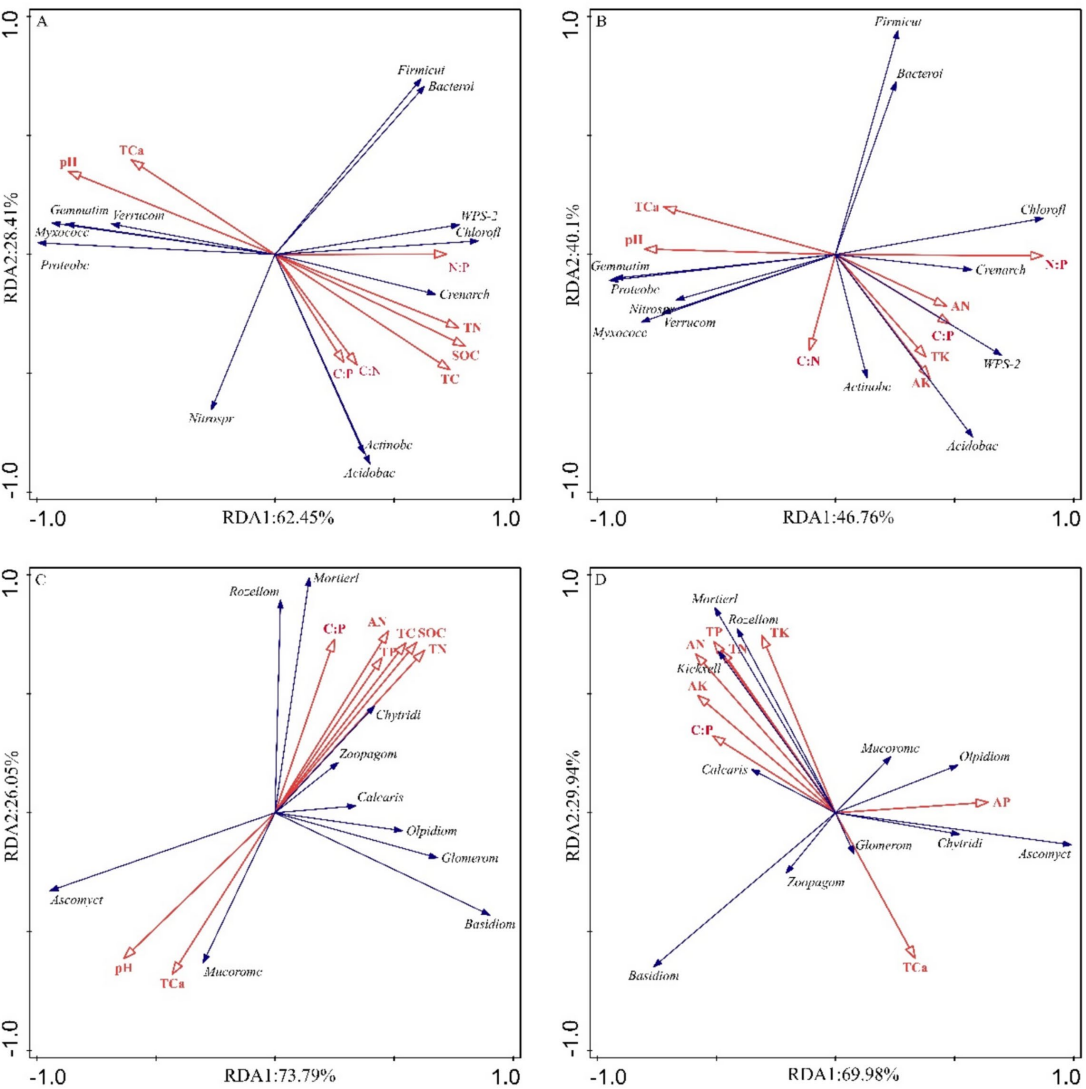
Redundancy analysis and correlation analysis between the microbial phyla and the soil physicochemical properties. **(A)** RDA of bulk soil bacterial communities and soil physicochemical variables. **(B)** RDA of rhizosphere soil bacterial communities and soil physicochemical variables. **(C)** RDA of bulk soil fungal communities and soil physicochemical variables. **(D)** RDA of rhizosphere fungal bacterial communities and soil physicochemical variables.

Mantel tests and redundancy analysis (RDA) demonstrated significant associations between soil physicochemical parameters and microbial community composition (Figures 8, 9; Table 1). In bulk soil systems, bacterial Shannon diversity exhibited significant associations with total potassium (TK) and stoichiometric ratios (C/N, C/P) (Figure 8A), whereas fungal Chao1 diversity demonstrated broader nutrient sensitivity, correlating with total carbon (TC), organic carbon (SOC), nitrogen species (TN, AN), phosphorus (TP), calcium (TCa), and pH (Figure 8B). In rhizosphere soil, bacterial alpha diversity was significantly linked to TK and N:P (Figure 8C), while fungal Shannon diversity was significantly correlated with pH (Figure 8D).

**Table 1:** The contributions of soil physicochemical properties to the variations for bacteria and fungi communities in the rhizosphere and bulk soils.

Soil properties	Bacteria				Fungi			
	Rhizosphere	*P*	Bulk	*P*	Rhizosphere	*P*	Bulk	*P*
	Explained (%)		Explained (%)		Explained (%)		Explained (%)	
pH	5.2	0.498	50.5	0.01			4.4	0.06
TC			17.1	0.07			2.8	0.35
TN			12.3	0.08	21.2	0.102	41.2	0.024
TP					5.3	0.276	6.1	0.174
TK	7.8	0.428			5.8	0.196		
TC_a_			7.3	0.1			4.5	0.272
SOC							9.3	0.14
AN	9.3	0.358			37.4	0.024	31.7	0.034
AP					1.7	0.644		
AK	14.8	0.224			3	0.424		
N/P	35.5	0.006	5.8	0.15				
C/N	13.8	0.096	5.8	0.042				
C/P	12.6	0.252	0.7	0.314	23.4	0.05		

Ordination analyses revealed differential explanatory power between soil compartments. For bacterial communities, the first two RDA axes explained 90.86% (bulk soil) and 86.86% (rhizosphere) of compositional variation (Figures 9A,B). Fungal communities demonstrated significantly greater axis explanatory power at 99.84% (bulk) and 99.92% (rhizosphere) (Figures 9C,D). Bacterial communities were predominantly structured by pH and stoichiometric ratios (C/N, C/P, N/P) (Figures 9A,B; Table 1), while fungal communities were more strongly affected by TN, TP, and AN (Figures 9C,D; Table 1). Notably, compartment-specific modulated these relationships: rhizobacterial composition were governed by nutrient availability (AN, AK) and elemental ratios (N/P, C/N, C/P), while bulk soil bacteria responded to pH and total nutrient pools (TC, TN) (Figures 9A,B; Table 1). Fungal biogeography exhibited similar patterns, with rhizosphere communities influenced by AN, TN, and C:P ratios, contrasting with bulk soil variations governed by TN, AN, and SOC (Figures 9C,D; Table 1).

## 4 Discussion

### 4.1 Differences in microbial alpha diversity and composition

Long-term reforestation significantly altered soil microbial alpha diversity and community composition across the chronosequence. Consistent with expectations, *P. armandii* plantation exhibited significant changes in soil microbial alpha diversity, aligning with observations in temperate and subtropical forests (Li et al., 2020; Yan et al., 2020). Bacterial and fungal Shannon diversity exhibited a distinct pattern: initially decreasing, then increasing with stand age. This dynamic primarily stems from shifting resource availability, changes in root exudation, and successional niche dynamics driven by plant community development (Wang et al., 2023). In young forests, harsh, variable conditions coupled with highly fluctuating resources favor r-selected microbial strategists (high reproductive rates, rapid growth, broad niches) (Liu W. et al., 2020). As plant biomass, root density, and competition increase during mid-succession, resource limitation intensifies: heightened plant and microbial nutrient uptake depletes pools, and shifts in root exudate composition and litter chemistry selectively favor fewer microbial taxa (Kasel and Bennett, 2007; Malchair and Carnol, 2009). Concurrently, successional niche dynamics occur, where early-successional specialists are replaced but stable, K-selected communities are not yet established, resulting in the observed diversity minimum (Zhang et al., 2016). Ultimately, mature forests provide stable conditions with predictable, diverse resource inputs (e.g., litterfall, complex exudates). This promotes K-selected strategists (high competitiveness, specialization) and allows for resource partitioning among microbes across a broader resource spectrum, leading to higher alpha diversity than mid-succession (Cui et al., 2018; Wang et al., 2023).

Crucially, rhizosphere and bulk soil exhibited divergent microbial diversity patterns. Rhizosphere soil generally had lower alpha diversity than bulk soil, except for fungal Chao1 diversity in young forests, which aligns with previous studies in grasslands and agricultural soils (Marilley and Aragno, 1999; Ai et al., 2012). This is likely attributed to strong host plant selection via root exudates (Liu L. et al., 2020), which create a distinct physicochemical microhabitat favoring specific microbial populations. This rhizosphere filtering, driven by plant-derived signals and organic compounds, shapes a community distinct from the bulk soil reservoir. Microhabitat heterogeneity is thus fundamentally higher in the rhizosphere. However, the persistent similarity between compartments underscores that the rhizosphere microbiome is shaped by both recruitment from the bulk soil reservoir and local plant-microbe feedbacks within the rhizosphere environment (Bram et al., 2017; Veach et al., 2019).

Microbial community composition varied significantly across stand ages (Figures 3, 4), driven primarily by shifts in the abundance of key bacterial and fungal phyla. These compositional shifts reflect functional adaptations associated with stand ages. Changes in bacterial composition were largely attributed to fluctuations in Proteobacteria, Acidobacteria, and Actinobacteria. The high abundance of Proteobacteria in young forests is consistent with their broad ecological niche and nitrogen fixation capabilities (Kim et al., 2021; Rojas et al., 2016), supporting early forest growth. In contrast, the increased abundance of Acidobacteria and Actinobacteria in middle-aged and mature forests aligns with Acidobacteria’s tolerance to nutrient-poor soils and Actinobacteria’s role in lignin decomposition (Kirby, 2005), facilitating conifer forest development under changing conditions. The decline in Proteobacteria abundance in middle-aged and mature forests may be linked to reduced nitrogen availability, favoring non-nitrogen-fixing bacteria (Zheng et al., 2020). Fungal community shifts were primarily driven by changes in the relative abundance of Ascomycota and Basidiomycota. A progressive enrichment of Basidiomycota and reduction in Ascomycota across the chronosequence indicates a transition in decomposition strategy: from preferential utilization of labile carbon compounds by Ascomycota towards the breakdown of recalcitrant complex polymers (e.g., lignin) dominated by Basidiomycota (Lodato et al., 2021). This functional shift has significant ecosystem implications: it enhances the formation of stable soil organic carbon pools through the accumulation of recalcitrant residues and microbial necromass, contributing to long-term carbon sequestration. Concurrently, the slower decomposition of complex compounds may regulate nutrient cycling rates, influencing nitrogen and phosphorus mineralization and availability within the ecosystem.

Principal Coordinates Analysis (PCoA) distinctly separated microbial communities into three groups corresponding to forest age classes (Figure 4), reinforcing the strong taxonomic dependence observed across the *P. armandii* chronosequence. Despite these significant temporal shifts in community structure, core bacterial and fungal phyla maintained relative stability throughout forest development. Furthermore, although rhizosphere filtering consistently differentiated the composition between rhizosphere and bulk soil compartments (Ning et al., 2022; Luo et al., 2021; Baudoin et al., 2003), we observed no significant temporal differences in the compositional divergence between these compartments across age classes. This finding further underscores the dual importance of bulk soil reservoirs and rhizosphere recruitment processes in shaping the microbial communities.

### 4.2 Microbial co-occurrence network in the bulk and rhizosphere soils

Soil microbial communities, like plant communities, exhibit complex interaction networks encompassing both mutualistic (e.g., symbiosis) and antagonistic (e.g., competition, predation) relationships (de Menezes et al., 2017). While co-occurrence network analysis does not directly reveal *in situ* interactions, it remains a valuable tool for elucidating microbial coexistence patterns in environmental samples (Barberán et al., 2012) and exploring links between ecosystem complexity and stability (Zhu et al., 2020). Our analysis revealed distinct co-occurrence network properties in rhizosphere versus bulk soil, indicating that differing niche conditions driven by plant roots sculpt unique bacterial and fungal interaction patterns. This differentiation is primarily attributed to plant root exudates, which alter nutrient availability, suppress pathogens, and recruit specific microbes (Chaparro et al., 2014; Zhao et al., 2021), demonstrating strong host-driven selection and metabolic influences. Furthermore, both forest successional stage and microhabitat significantly modulated rhizosphere network structure, highlighting the synergistic effects of plant development and environmental filtering.

Notably, rhizosphere networks exhibited significantly higher topological complexity (e.g., positive edge proportion, clustering coefficient, average connectivity, and density) than bulk soil networks (Figure 6; Supplementary Table S1). This increased complexity, potentially reflecting greater species interactions (e.g., commensalism, syntrophy, mutualism) and niche overlap (Yuan et al., 2021), may support enhanced community stability and resilience against environmental fluctuations. Three mechanisms likely underpin this complexity: (1) microenvironmental modifications induced by roots (e.g., hydrologic shifts) (Wang et al., 2018); (2) enhanced diversity of potential interactions, including cascading effects (Shi et al., 2016); and (3) exudate-mediated stimulation of microbial exchanges via carbon substrates (e.g., organic acids, sugars, amino acids) (Philippot et al., 2013).

Central to understanding the ecological implications of these networks are keystone taxa, identified based on their topological roles (e.g., connectors, module hubs), which are hypothesized to disproportionately influence network stability and key ecosystem functions such as soil organic matter (SOM) metabolism and nitrogen (N) cycling (Xun et al., 2021). Our analysis identified keystone bacterial taxa primarily from Proteobacteria, Actinobacteria, Ascomycota, and Mortierellomycota, and fungal keystones from Firmicutes, Chloroflexi, and Bacteroidota, serving as critical connectors and module hubs. The ecological dominance of these keystones stems from physiological adaptations (e.g., high-affinity transporters in bacteria) (Ward et al., 2009) and functional versatility crucial for ecosystem processes (e.g., lignocellulose degradation and mycoparasitism in fungi) (Wang et al., 2023). Environmental selection in forests, favoring taxa adept at utilizing complex organic polymers abundant in detritus (e.g., chitin, phospholipids) (Chen et al., 2019), further shapes their prominence. Crucially, the differences in keystone species composition and function between rhizosphere and bulk soil reflect the interplay of vegetation development and soil properties, directly linking these network hubs to spatial variations in nutrient cycling potential. In summary, during plantation development, the versatile metabolisms of these keystone species likely contribute significantly to shaping community structure and mediating interspecies interactions that underpin ecosystem functions (Fan et al., 2018).

However, it is essential to acknowledge the limitations inherent in co-occurrence network inference from sequencing data. These networks represent statistical associations (co-occurrence/co-exclusion patterns), not confirmed biological interactions. Factors like shared environmental preferences or dispersal limitations can generate patterns indistinguishable from direct interactions. Therefore, the inferred interactions (mutualism, competition, etc.) and the direct functional roles of “keystone” taxa based solely on topology remain hypotheses requiring further validation (e.g., through targeted experiments, cultivation, or functional genomics). Our interpretations regarding stability, resilience, and specific functional contributions (e.g., to nutrient cycling rates) are thus cautious extrapolations based on network theory and identified taxa, avoiding claims of direct mechanistic proof.

### 4.3 Microbial community assembly dynamics

Deciphering the mechanisms governing microbial community assembly remains a central challenge in microbial ecology (Zhou and Ning, 2017). In our study, deterministic processes were found to drive bacterial community assembly during the development of *P. armandii* plantations in both rhizosphere and bulk soils, aligning with previous research (Xu et al., 2022; Zhao et al., 2018). This deterministic dominance in bacteria can be attributed to several key drivers: (1) Their rapid generation times (Sun et al., 2017) and higher transmission rates (Schmidt et al., 2014) enable swift responses to environmental gradients such as shifts in soil pH, moisture, or nutrient availability (Rinnan et al., 2007; Ren et al., 2021). (2) Crucially, biotic interactions, particularly with the host plant, exert strong selective pressures. This is especially pronounced in the rhizosphere, where deterministic processes were significantly stronger than in bulk soil. The primary driver of this rhizosphere effect is the concentrated flux of diverse root exudates (e.g., carbon substrates, nitrogenous compounds, flavonoids, salicylic acid, phytoalexins) (Zhu et al., 2016; Vieira et al., 2020). These exudates create a distinct chemical environment gradient that selectively enriches microbial taxa phylogenetically or functionally adapted to utilize these resources, often enhancing nutrient cycling or promoting plant growth (Zhang et al., 2019). In contrast, the attenuated chemical gradient and weaker plant-driven biotic interactions in bulk soil result in comparatively weaker deterministic filtering.

Conversely, fungal community assembly exhibited stronger stochasticity, implying weaker environmental filtering overall and highlighting the importance of other drivers. This aligns with growing recognition of neutral processes in mycobiome organization (Wang et al., 2016). The enhanced stochasticity in fungi likely stems from inherent biological traits imposing constraints: (1) Limited dispersal capacity, associated with larger propagule sizes (Chen W. et al., 2020; Chen W. M. et al., 2020), particularly for specialists like symbionts or biotrophs dependent on spatially constrained hosts or resources (Wardle, 2006; Wang et al., 2022), reduces their ability to track environmental gradients efficiently. (2) Multicellular growth strategies enhance substrate exploitation but inherently limit mobility, further amplifying dispersal limitation and rapid response to environmental gradients across larger spatial scales (Heaton et al., 2020). These observations support theoretical frameworks linking microbial traits to assembly, such as the “size plasticity” (Farjalla et al., 2012) and “size-dispersal tradeoff” hypotheses (Shurin et al., 2009). Consequently, dispersal limitation and drift (random birth/death events) become more critical drivers than fine-scale environmental filtering or intense biotic selection for fungal communities. The specificity of many fungal interactions (e.g., host-pathogen or mycorrhizal symbioses) can also create patchy resource distributions, further amplifying the role of stochastic processes like dispersal limitation in assembly.

### 4.4 Effects of soil environmental factors on microbial communities

This study confirms that the structure of soil microbial communities is intricately regulated by multiple environmental factors, and this regulation varies depending on microbial groups (bacteria vs. fungi) and their specific soil microhabitats (rhizosphere vs. bulk soil). Bacterial community composition was primarily governed by soil pH (Chen W. et al., 2020; Chen W. M. et al., 2020) and key elemental stoichiometric ratios (C/N, C/P, N/P) (Delgado-Baquerizo et al., 2019; Cao et al., 2010; Dini-Andreote et al., 2014), supporting the prevailing view that pH acts as a critical filter for bacterial diversity and that stoichiometric ratios impose fundamental constraints on bacterial metabolism and community structure. Within the rhizosphere microhabitat shaped by root activity, bacterial composition was mainly driven by available nutrients (AN, AK) and elemental stoichiometric ratios (N/P, C/N, C/P), reflecting the selective effects of root exudates in enhancing nutrient availability and altering substrate stoichiometry. In contrast, bacterial communities in the bulk soil responded more strongly to factors reflecting fundamental soil conditions and total nutrient pools, such as pH, TC, and TN.

Conversely, fungal community composition exhibited a stronger response to total nutrient pools (TN, TP) and AN, likely related to their diverse roles in nutrient cycling (e.g., saprotrophic, symbiotic fungi) and acquisition strategies. The specific driving factors also differed by microhabitat. Rhizosphere fungal communities were primarily associated with AN, TN, and the C:P ratio, underscoring the importance of nitrogen (both total and available forms) and carbon-phosphorus balance in the root-influenced zone (Li D. D. et al., 2018; Li J. et al., 2018; Wang et al., 2019). In contrast, variation in bulk soil fungal communities was mainly driven by TN, AN, and SOC in combination, indicating that their structure is simultaneously influenced by nitrogen status and organic carbon availability (a key energy source for saprotrophic fungi) (Li D. D. et al., 2018; Li J. et al., 2018; Wang et al., 2019).

Collectively, these findings deepen our understanding of the mechanisms shaping soil microbial biogeographical patterns. They highlight the necessity of considering spatial heterogeneity (particularly the rhizosphere effect) and the specificity of microbial functional groups when studying the relationships between microbial communities and their environment.

## 5 Conclusion

This study elucidated the temporal dynamics and drivers of microbial community assembly in rhizosphere and bulk soil across different stand age of *P. armandii* plantations in a karst ecosystem. Key findings demonstrated significant stage-dependent variations in microbial *α*-diversity, community structure, and functional profiles for both bacteria and fungi, while highlighting the higher network complexity and interconnectivity within the rhizosphere compared to bulk soil. Crucially, the assembly processes exhibited domain specificity, with bacterial communities predominantly governed by deterministic selection and fungal communities by stochastic processes, the influence of soil compartment varying temporally. Multivariate analyses consistently identified soil physicochemical properties as the primary regulator of these microbial patterns throughout plantation development. These results have critical implications for karst ecosystem restoration and soil health management: understanding the dominant role of soil properties and the temporal dynamics informs targeted interventions to manipulate microbial communities for enhancing plant establishment and soil fertility in degraded karst landscapes. Future research should prioritize: (1) long-term monitoring to link microbial dynamics with restoration outcomes, (2) functional validation of key microbial groups identified in network analyses, and (3) integrating multi-omics approaches to unravel plant-microbe-soil feedbacks driving succession in fragile karst systems.

## Data Availability

The data presented in the study are deposited in the NCBI SRA repository, accession number PRJNA1063200 and PRJNA1132892.
